# Poly(2‐Oxazoline)‐Based Polyphotoacids: Synthesis, Solution Behavior, and Cellular Uptake of Multi‐Responsive Intracellular Transporters

**DOI:** 10.1002/marc.202500455

**Published:** 2025-10-16

**Authors:** Leonid I. Kaberov, Amod Godbole, Laura Klement, Sreevalsan Achikkulathu, Avinash Chettri, Benjamin Dietzek‐Ivanšić, Carsten Hoffmann, Felix H. Schacher

**Affiliations:** ^1^ Institute of Organic Chemistry and Macromolecular Chemistry (IOMC) Friedrich‐Schiller‐University Jena Jena Germany; ^2^ Jena Center For Soft Matter (JCSM) Friedrich‐Schiller‐University Jena Jena Germany; ^3^ Institut für Molekulare Zellbiologie CMB – Center for Molecular Biomedicine; Universitätsklinikum Jena Friedrich‐Schiller‐University Jena Jena Germany; ^4^ Institute of Physical Chemistry and Abbe Center of Photonics Friedrich‐Schiller‐University Jena Jena Germany; ^5^ Department of Functional Interfaces Leibniz Institute of Photonic Technology Jena e.V. Jena Germany; ^6^ Center for Energy and Environmental Chemistry (CEEC) Friedrich‐Schiller‐University Jena Jena Germany

**Keywords:** cellular uptake, excited state proton transfer, light‐responsive, multi‐stimuli‐responsive, photoacid, poly(2‐oxazoline)

## Abstract

This work describes the first polymeric photoacids based on poly(2‐oxazoline)s. The presence of 1‐hydroxypyrene units—a photoacid that can increase its acidity upon blue light irradiation through excited‐state proton transfer—allows remote control over material polarity and charge. A set of multi‐stimuli‐responsive polymers was obtained via partial hydrolysis of poly(2‐ethyl‐2‐oxazoline) precursors, followed by functionalization with 6/8‐acetoxypyrene‐1‐sulphonyl chloride. By varying the reagent ratio, complete or partial functionalization of hydrolyzed units can be achieved; in the latter case some remaining polyethyleneimine (PEI) groups can also be realized. Conducted cytotoxicity studies show high viability of selected cell lines, which serves as a first reported confirmation of biocompatibility of polyphotoacids. This was further confirmed via cellular uptake experiments, where the synthesized copolymers show no cell damage after 24 h of incubation. Apart from that, the polyphotoacid with the highest hydroxypyrene content and remaining PEI‐units shows the fastest uptake. The same terpolymer also shows a high capacity for hydrophobic drug encapsulation. The obtained results support the prospect of polyphotoacids as materials in the context of intracellular transport.

## Graphical abstract

The ability of photoacids to increase their acidity in response to light irradiation (excited state proton transfer, ESPT) makes them attractive building blocks for the design of stimuli‐responsive copolymers. In this contribution, we present highly biocompatible polyphotoacids based on poly(2‐oxazoline)s bearing 1‐hydroxypyrene units. Low cytotoxicity, fast cellular uptake, and high loading capacity highlight their potential for drug delivery.

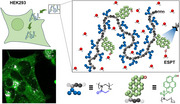

## 1 Introduction

The exploration of synthetic polymers in biomedical applications is mainly driven by the broad possibilities for adjusting functionality, architecture, and targeting moieties [1, 2, 3]. For use as biomaterials, these polymers must meet certain criteria, such as being non‐toxic and biocompatible [4, 5]. Traditionally, due to stealth characteristics and biocompatibility, many examples for polymeric biomaterials are based on poly(ethylene glycol) (PEG) [6, 7]. However, within the last years, limitations of commonly used PEG‐based biomaterials have become more and more evident: it is non‐biodegradable, show non‐specific interactions with blood, and can cause hypersensitivity, allergic reactions, or accelerated blood clearance—in addition to an overall limited functionality. These drawbacks encouraged researchers to develop alternative polymeric materials to overcome these constraints [8, 9, 10].

In this context, poly(2‐oxazoline)s (POx) stand out as a promising alternative to PEG‐based biomaterials [11, 12]. POx have been known for over five decades and were first synthesized via living cationic ring‐opening polymerization (CROP) of 2‐substituted‐2‐oxazoline monomers [13]. As a biomaterial, POx has gained traction due to its rich chemistry, straightforward synthesis, and the ability to tailor properties such as molar mass, hydrophilic‐hydrophobic balance, and architecture [14, 15]. However, the widespread availability of PEG caused POx to fall into oblivion [14]. Recent studies on POx show that for some application fields, these materials even have advantages if compared to PEG [16, 17, 18]. This has led to several studies addressing a comparative analysis of PEG, POx, and the respective conjugates, revealing similar cytotoxicity and hemolytic activity in a broad concentration range [12, 19, 20, 21, 22]. At the same time, several studies confirm higher stability of POx against oxidative degradation, allowing it to preserve function and structure more effectively in the presence of reactive oxygen species [22, 23, 24].

In addition, POx can be functionalized in the side chain to impart response toward changes in pH, temperature, ionic strength of the solution, or enzymatic activity, which creates versatile adaptive materials [25]. These materials, in particular, show potential in biomedical applications as carriers enabling triggered delivery of drugs, DNA, or genes [26, 27, 28].

Light‐responsive polymers undergo changes when exposed to light of different wavelengths. Depending on the photochemical properties, the light‐sensitive functional groups can trigger dimerization, isomerization, and/or cleavage in the polymer, which causes changes in physical properties or morphological conversion, such as cross‐linking, dissociation, or changes in shape [29, 30]. Photo‐responsive polymer systems can be developed by introducing functional groups, such as derivatives of azobenzene, stilbene, and spiropyrane, which can induce reversible isomerization upon exposure to light [31].

Even though many advancements for biomedical applications have been achieved with single stimuli‐responsive polymers, the ability of such systems to overcome biological barriers (mononuclear phagocyte system, cellular uptake, and endosome escape) is limited. Multi‐stimuli responsive drug‐loaded polymer nanoparticles are capable of more effective transition through the biological barriers, which, in the end, determines the effectiveness of the therapy [32]. Such materials are of great interest for biomedical applications, especially for cancer treatment [33, 34, 35, 36, 37].

Temperature and light are physical stimuli applicable externally to any system, and light can even be controlled spatiotemporally [38]. Therefore, incorporation of photoswitches provides an additional leverage on the conformational behavior of thermoresponsive macromolecules. In a few works addressing dual light/temperature responsive poly(2‐oxazoline)s, azobenzene units have been employed as the light responsive component. Jim et al. developed an azobenzene end‐capped thermo‐responsive poly(2‐*iso*‐propyl‐2‐oxazoline) (P*i*PrOx), where measured values for LCST / cloud point temperatures were shown to depend on the azobenzene switching state [39]. Using *i*PrOx‐containing terpolymers and spiropyrane‐based crosslinkers, Weck and co‐workers designed and studied the effectivity of shell cross‐linked micelles as catalytic nanoreactors with light and temperature controlled selectivity [40]. In our own work of Wang et al., the introduction of azobenzene units in the side chain of poly(2‐ethyl‐2‐oxazoline) (PEtOx) allowed light‐mediated control over both cloud point and nanoparticle size in aqueous solutions [41, 42]. However, the isomerization of azobenzene units does not provide a significant shift of cloud point temperatures for thermoresponsive polymers, prompting the search for new chromophores [43].

Recently, we introduced polyphotoacids based on polymerizable naphthols, leading to dual light and pH‐sensitive copolymers and block copolymers [44]. The concept is based on the ability of naphthol to undergo excited state proton transfer (ESPT)—a significant increase of acidity in response to UV irradiation—which in turn affects the polarity of the photoacid. The light‐mediated variation of the proton concentration using photoacids and photoacid generators (PAG) has demonstrated potential in a wide range of biomedical applications, including photo‐triggered activation of enzymes, analysis of protein binding sites, or tumour and wound treatment [45, 46, 47, 48]. By varying the monomer structure, we were able to adjust the *pK*_a_/*pK*_a_* and photostability of the resulting polyphotoacids [49, 50]. When incorporated into the hydrophobic block of an amphiphilic block copolymer, photoacid units could induce the swelling of the micellar core in response to irradiation with light [49, 51]. The expansion of the aromatic π‐system when changing from naphthol to pyrenol enables the induction of the ESPT with visible light (405 nm) irradiation, which makes polyphotoacids prospective for the fabrication of drug delivery systems [52]. Here we report on synthesis, solution behavior, and cellular uptake studies of the first poly(2‐oxazoline)‐based polyphotoacids, which can be activated in the visible light range.

## 2 Results and Discussion

### 2.1 Polymer Synthesis and Characterization

**SCHEME 1:**
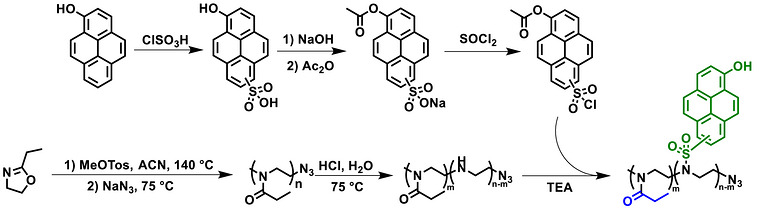
Synthesis of poly(2‐oxazoline)‐based polyphotoacids.

For the preparation of the targeted polyphotoacids, partial hydrolysis of poly(2‐oxazoline) homopolymers with subsequent functionalization was used (Scheme 1). This allows adjusting the content of photoacid in the final material with high precision.

The hydroxypyrene‐based modification agent – 6/8‐acetoxypyrene‐1‐sulphonyl chloride (AcOPyrSO_2_Cl)—was synthesized according to the procedure described in previous work with minor modifications [52].

**FIGURE 1:**
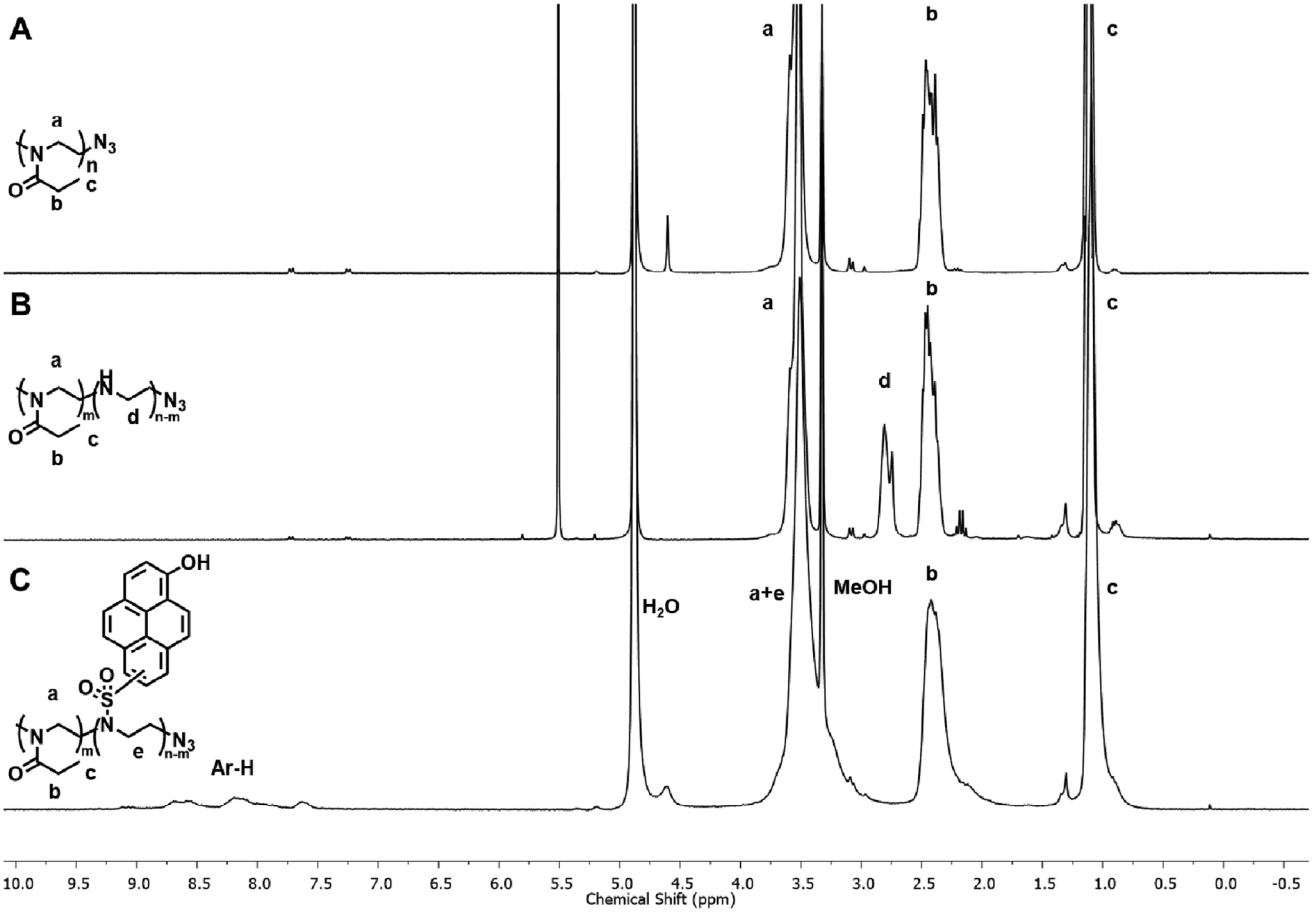
Stacked ^1^H‐NMR spectra of the PEtOx homopolymer (A), and the respective P(EtOx_0.84_‐*co*‐EI_0.16_) (B), and P(EtOx_0.84_‐*co*‐(HOPyr)SAz_0.16_) (C) copolymers in CD_3_OD.

The poly(2‐ethyl‐2‐oxazoline) homopolymer was synthesized according to a standard protocol described elsewhere [53]. During the second step, poly(2‐ethyl‐2‐oxazoline)‐*co*‐poly(ethylene imine) (PEtOx‐*co*‐PEI) was obtained via acidic hydrolysis at 75°C. The duration of the hydrolysis was set to 50, 100, 200, and 300 min, which results in a stepwise increase of the fraction of PEI units as could be seen from the NMR (Figure 1B). The final step was the sulfonylation of PEtOx‐*co*‐PEI with AcOPyrSO_2_Cl in the presence of a base. The successful synthesis of the targeted polyphotoacid was confirmed by the absence of PEI signals at 2.75 ppm and the appearance of aromatic peaks in NMR. The final material represents a statistical copolymer containing 2‐oxazoline and sulfonamide units. To simplify the nomenclature, the obtained copolymers will be named according to the “original monomer” name, i.e., poly[(2‐ethyl‐2‐oxazoline)‐co‐(1‐(6/8‐hydroxypyrene)sulphonylaziridine)] or P(EtOx‐*co*‐(HOPyr)SAz) (Table 1).

**TABLE 1:** Characteristics of the herein synthesized poly(2‐oxazoline)s.

Composition^a^	Hydrolysis time	*M*_n_ (g/mol)^b^	*Đ* ^b^	*pK*_a_	*pK*_a_^*^ ^c^
P(EtOx_0.97_‐*co*‐(HOPyr)SAz_0.03_)	50 min	10100	1.08	8.2	1.8
P(EtOx_0.95_‐*co*‐(HOPyr)SAz_0.05_)	100 min	10000	1.09	8.2	1.8
P(EtOx_0.89_‐*co*‐(HOPyr)SAz_0.11_)	200 min	11200	1.11	8.5	2.1
P(EtOx_0.76_ *‐co*‐(HOPyr)SAz_0.24_)	300 min	12700	1.12	—	—
P(EtOx_0.77_‐*co‐PEI_0.06_‐co*‐(HOPyr)SAz_0.17_)	300 min	12900	1.14	8.8	2.4

^a^ obtained by ^1^H NMR (300 MHz, CD_3_OD);

^b^ obtained by SEC (DMAc + 0.21 wt.% LiCl);

^c^ obtained by Förster cycle analysis.

Apart from the described procedure, where the excess of the protected pyrenolsulfonylchloride was used to ensure complete conversion of PEI, we performed the functionalization of PEtOx_0.77_‐*co*‐PEI_0.23_ with a deficiency of AcOPyrSO_2_Cl. With that, a small amount of cationic PEI units can promote cellular uptake due to a partial positive charge of the resulting copolymers. The NMR spectrum of P(EtOx‐*co‐PEI‐co*‐(HOPyr)SAz) contains a signal at 2.8 ppm (Figure S1), indicative of the remaining CH_2_‐groups of PEI. Due to the broadening of the signals in NMR, it is difficult to determine the exact fraction of remaining PEI. A rough estimation based on the comparison of the intensities of the peaks provides the polymer composition as P(EtOx_0.77_‐*co*‐PEI_0.06_‐*co*‐(HOPyr)SAz_0.17_).

### 2.2 UV/Vis Spectroscopic Studies

**FIGURE 2:**
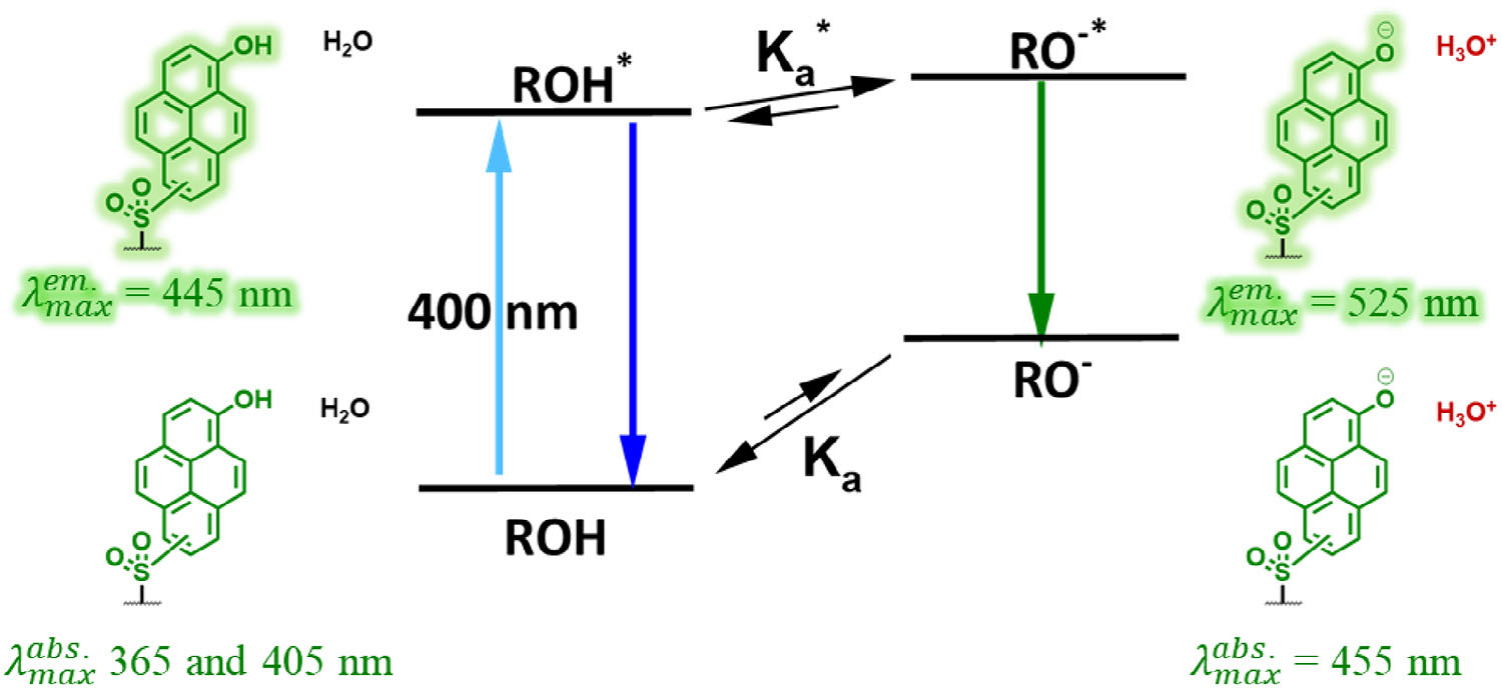
A simplified scheme of an excited‐state proton transfer (ESPT) process for pyrenol‐based polyphotoacids.

Upon excitation with light, a photoacid can undergo excited‐state proton transfer (ESPT), leading to a significantly higher acidity compared to the ground state (Figure 2) [54]. The excited protonated species (ROH^*^) transfers a proton to an H‐bonded water molecule, forming an excited deprotonated species (RO^−*^), which emits at a wavelength distinct from the emission wavelength of ROH^*^.

Figure S2 shows the steady‐state absorption and emission spectra of selected copolymers in 4 m HCl, as well as at pH 11.0 and pH 5.3. In 4 m HCl, none of the polymeric photoacids participate in ESPT. Therefore, the absorption and emission spectra are pure signatures of ROH ($\lambda_{max}^{abs.}$ 365 and 405 nm) and ROH^*^ ($\lambda_{max}^{em.}$ = 445 nm) [55, 56]. On the other hand, at pH 11.0 the molecules in the ground‐state are predominantly RO^−^ ($\lambda_{max}^{abs.}$ = 455 nm—red‐shifted compared to protonated species) whereas the emission is associated with RO^−*^ ($\lambda_{max}^{em.}$ = 525 nm) [55, 56]. At an intermediate pH of 5.3, the absorption spectrum of the polyphotoacids features maxima at 365 and 405 nm, which is identical to the spectrum recorded in 4 M HCl establishing that ROH is the predominant species in the ground state at pH 5.3. Interestingly, for all polymers a dual emission is observed with maxima at 445 nm (ROH^*^) and 525 nm (RO^−*^), indicating ESPT is at least partially operative. The presence of the emission band at 445 nm indicates that ESPT is not complete, and an equilibrium exists between the two species.

**FIGURE 3:**
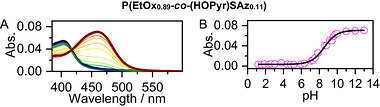
(A) Steady‐state absorption spectra of P(EtOx_0.89_‐*co*‐(HOPyr)SAz_0.11_) in buffer solutions with pH values ranging from 1.0 to 13.0. (B) Absorbance versus pH plot at 455 nm. All experiments are conducted in non‐inert (aerated) conditions. The figures are adapted from previous work [57]. Please note that the value of the emission maximum of ROH* species provided in the main text has been wrongly reported earlier (Page 4, section “Steady‐state absorption and emission spectroscopy ofHPSAPMA and I(a–d)”)—the correct value is $\lambda_{max}^{em.}$ = 445 nm, as determined from the emission spectra.

To determine the *pK*_a_ of polyphotoacids pH‐metric UV‐visible absorption titration was conducted. With an increase in the pH of the buffer solution, the band at 405 nm, which corresponds to ROH decreases with a concomitant increase in the absorption band at 455 nm, which is a signature of RO^−^ (Figure 3A). A plot of the absorbance at 455 nm versus pH yields a sigmoidally shaped curve (Figure 3B). The pH value at which the concentrations of ROH and RO^−^ are equal is the *pK*_a_, which is the pH corresponding to the mean absorbance (obtained by taking the mean of the highest and the lowest absorbance values from the plot). An increase in the *pK*_a_ from 8.2 to 8.8 is observed for the obtained copolymers. This decrease in the ground state acidity is a result of an increased hydrophobicity of the copolymers due to an increase in pyrenol content. We hypothesize that an overall increase in hydrophobicity introduces sub‐domains in the copolymer, which may not have access to water molecules, thereby limiting the acidity [57]. The *pK*_a_^*^ for the copolymers is determined using Förster cycle analysis [58]. For all the polymeric photoacids Δ*pK*_a_ = *pK*_a_*—*pK*_a_ = −6.4, indicating an increased acidity in the excited state compared to the ground state. However, a slight increase in the *pK*_a_^*^ from 1.8 to 2.4 with an increase of pyrenol content is observed, demonstrating that the increased hydrophobicity introduced by increasing the pyrenol content also affects the acidity of the polymeric photoacids in the excited state. Apart from these slight variations, overall, the amphiphilic copolymers show a distinct increase in acidity upon irradiation with light.

### 2.3 Stimuli‐Responsive Behavior

The temperature‐dependent solubility of PEtOx‐based homo and block copolymers in aqueous media is well known [41, 42, 59, 60]. Furthermore, besides the adjustment of the *T*_cloud_ by copolymerization of EtOx with other 2‐oxazolines, the incorporation of pH‐ or light‐responsive comonomers is widely used to control material properties. Pyrenol in its electronic ground state can be regarded as a hydrophobic side chain. However, upon optical excitation, ESPT can take place, and upon the formation of pyrenolate anions the hydrophilicity will increase, and this will affect any cloud point temperature of such aqueous solutions. Besides that, the deprotonation of pyrenols could be achieved by varying the solution pH. From that point of view, the photoacid units have an advantage in the sense that their polarity could be affected by both light and changes in pH [52].

**FIGURE 4:**
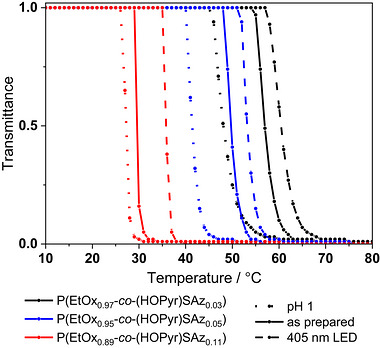
Transmittance versus temperature plots for the polyphotoacids in aqueous solutions: solid lines—as prepared, non‐irradiated; dotted lines—in acidic (pH 1) conditions; dashed lines—irradiated. C = 5 mg/mL.

These assumptions were confirmed by turbidimetry studies. As could be seen from Figure 4, in the “as prepared” state the aqueous solutions of P(EtOx_0.97_‐*co*‐(HOPyr)SAz_0.03_), P(EtOx_0.95_‐*co*‐(HOPyr)SAz_0.05_), P(EtOx_0.89_‐*co*‐(HOPyr)SAz_0.11_) exhibit temperature‐dependent solubility with cloud points in the range of 30–60°C, depending on the fraction of the sulfoaziridine units. Decreasing the pH value and, thus, protonation of the photoacid units in the copolymer significantly decreases *T*_cloud_, and the severity of this effect is reduced for the P(EtOx_0.89_‐*co*‐(HOPyr)SAz_0.11_) with the highest photoacid fraction. At the same time, under basic conditions, the corresponding copolymers are more hydrophilic (higher charge density), and for the solutions of P(EtOx_0.95_‐*co*‐(HOPyr)SAz_0.05_) and P(EtOx_0.89_‐*co*‐(HOPyr)SAz_0.11_) no cloud point could be observed up to 85°C already at pH 8 (Figure S5). The deprotonation of pyrenol units induced by blue light irradiation (405 nm), in turn, results in an increase of about 3–5°C for *T*_cloud_.

Exceptional behavior was observed for P(EtOx_0.77_‐*co*‐PEI_0.06_ *‐co*‐(HOPyr)SAz_0.17_), where no temperature‐dependent solubility was observed at any pH value, which we attribute to protonation of remaining PEI units at low pH, also leading to an increased hydrophilicity.

### 2.4 Self‐Assembly in Aqueous Solution

**FIGURE 5:**
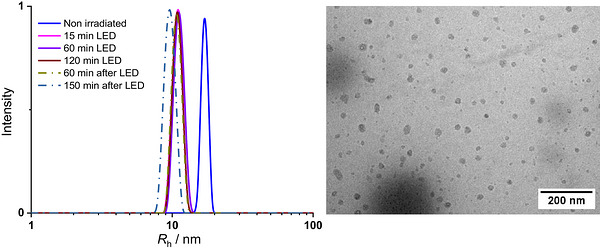
Distribution functions of hydrodynamic radius R_h_ of aqueous solutions of P(EtOx_0.77_‐*co*‐PEI_0.06_‐*co*‐(HOPyr)SAz_0.17_) (left) and respective TEM micrograph of the aggregates (right). C = 5 mg/mL.

The aggregation of the obtained copolymers in aqueous solution was investigated by dynamic light scattering, revealing that most compositions were molecularly dissolved. The hydrodynamic radius *R*_h_ for P(EtOx_0.77_‐*co*‐PEI_0.06_ *‐co*‐(HOPyr)SAz_0.17_) in aqueous solution was *R*_h_ ≈ 17 nm (Figure 5). Further investigation of this sample by transmission electron microscopy (TEM) revealed the presence of spherical aggregates, which we interpret as loose micelles [41, 52]. Interestingly, the copolymer featuring a higher pyrenol content is molecularly dissolved at comparable concentrations, which indicates that this aggregation is not only driven by hydrophobic interactions. According to literature, the *pK*_a_ values of diethylamine (*pK*_a_H = 11.2) and LPEI (*pK*_a_H ≈ 9, exact value depends on the neighbouring groups) are slightly higher than the ground state *pK*_a_ of 1‐hydroxypyrene units. This could result in a partial transfer of the proton from a PyrOH group to adjacent PEI repeat units, forming PyrO^−^ and PEI‐H^+^ interpolyelectrolyte complexes. However, such an assumption requires further in‐depth studies.

The irradiation of this solution by visible light (LED 405 nm) for 15 min resulted in a significant decrease of the *R*_h_ to ∼11 nm. Following irradiation for 2 h, as well as subsequent removal of the light source (for 1 h) had no significant effect on *R*_h_. The change in the size of nanoparticles in aqueous solution under light exposure should be attributed to the increased acidity of hydroxypyrene units in the excited state. This, in turn, results in changes of the hydrophilic‐hydrophobic balance and potential restructuring of the aggregates.

### 2.5 Encapsulation of Curcumin

Amphiphilic copolymers are widely used as a tool for encapsulation and transport of bioactive molecules [61]. Response to external triggers and eventual changes in aggregate structure or stability provides a powerful control mechanism for the release of encapsulated cargo. To estimate the ability of the herein obtained polyphotoacids to solubilize hydrophobic drugs, encapsulation experiments with curcumin as a model anticancer drug were performed. The copolymer‐curcumin systems were prepared by solvent exchange (nanoprecipitation) from methanol to water with a targeted polymer concentration of 3 mg/mL [62]. Any non‐solubilized curcumin was removed by centrifugation, and the loading capacity was controlled by UV–vis spectroscopy and high‐performance liquid chromatography (Figure S4). Here, P(EtOx_0.95_‐*co*‐(HOPyr)SAz_0.05_) and P(EtOx_0.89_‐*co*‐(HOPyr)SAz_0.11_) exhibited a rather poor loading capacity, whereas P(EtOx_0.77_‐*co*‐PEI_0.06_‐*co*‐(HOPyr)SAz_0.17_) with the highest pyrenol content was able to encapsulate around 48 mg per gram of copolymer (Table 2), which is comparable with previously reported data for Pox‐based block copolymers [62]. We attribute the higher drug loading for P(EtOx_0.77_‐*co*‐PEI_0.06_‐*co*‐(HOPyr)SAz_0.17_) to the formation of nanoparticles, which provides more efficient solubilization of hydrophobic curcumin.

**TABLE 2:** Curcumin load of the polyphotoacids.

Polymer composition	Curcumin load, mg/g^a^
P(EtOx_0.95_‐*co*‐(HOPyr)SAz_0.05_)	7.3
P(EtOx_0.89_‐*co*‐(HOPyr)SAz_0.11_)	8.7
P(EtOx_0.77_‐*co‐PEI_0.06_‐co*‐(HOPyr)SAz_0.17_)	48

^a^ UV–vis spectroscopy, absorption at 425 nm.

### 2.6 Cellular Uptake and Cytotoxicity

**FIGURE 6:**
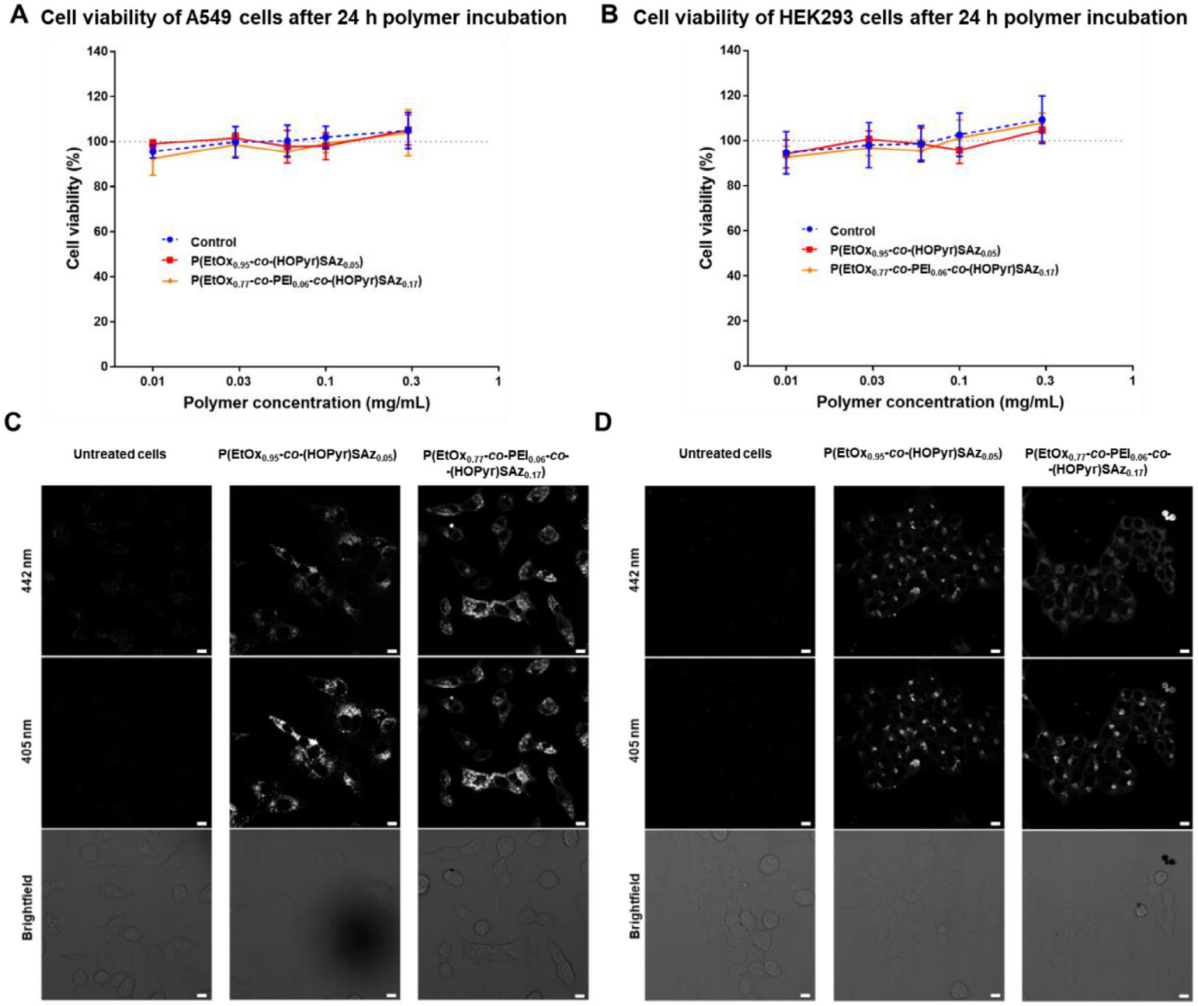
Cytotoxicity of P(EtOx_0.77_‐*co*‐PEI_0.06_‐*co*‐(HOPyr)SAz_0.17_) and P(EtOx_0.95_‐*co*‐(HOPyr)SAz_0.05_) in (A) A549 and (B) HEK293 cells (control—DMEM with 1% P/S). Confocal laser scanning microscope images of living (C) A549 or (D) HEK293 cells incubated with 0.1 mg/mL of the copolymers in DMEM with 1% P/S for 24 h with excitation at 442 nm (top panel), or 405 nm (middle panel) and emission in the range of 450–527 nm, and corresponding images using transmission light (bottom panel, brightfield). The scale bar represents 10 µm.

To probe any cytotoxic effects of the copolymers in general, A549 and human embryonic kidney (HEK293) cells were subjected to varying copolymer concentrations for 24 h, followed by evaluation of cell viability. Across all concentrations tested, ranging from 0.01 mg/mL up to 0.3 mg/mL, the copolymers led to observed cell viabilities exceeding 90% for both cell lines. In accordance with DIN EN ISO 10993–5 standards, which define cytotoxicity as a cell viability of ≤70%, the examined copolymers demonstrated non‐cytotoxic behavior up to a concentration of 0.3 mg/mL (Figure 6A,B). Live‐cell imaging confirmed the internalization of both copolymers by the cells within 24 h of incubation. Additionally, the copolymers did not induce any observable alterations in cellular morphology (Figure 6C,D).

**FIGURE 7:**
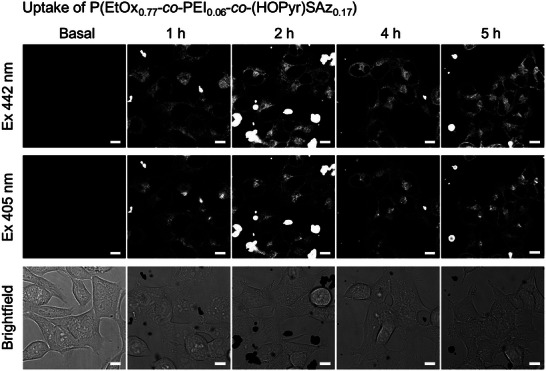
Uptake of P(EtOx_0.77_‐*co*‐PEI_0.06_‐*co*‐(HOPyr)SAz_0.17_) in HEK293 cells. Polymer concentration 0.1 mg/mL. The scale bar represents 10 µm for each panel.

To monitor the uptake of copolymers in living cells, we incubated living HEK293 cells with all the copolymers for the indicated time points (Figure 7). Images were acquired upon excitation at 405 and 442 nm and emission in the range of 450–527 nm. Most of the P(EtOx_0.77_‐*co*‐PEI_0.06_ *‐co*‐(HOPyr)SAz_0.17_) seem to be taken up by the cells within 1 h. We also observed some aggregates of this copolymer, albeit outside the cells. The remaining copolymers were not found to be taken up by the cells within 5 h (Figure S6). However, significant uptake of these copolymers could be achieved at longer incubation times (24 h), as can be seen from Figure 6C,D.

## 3 Conclusions

We report on the first poly(2‐oxazoline)‐based polyphotoacids via a sequential CROP—hydrolysis—functionalization synthetic strategy. Spectroscopic studies confirmed that the obtained polyphotoacids undergo excited‐state proton transfer with a Δ*pK*_a_ of up to 6.4. The obtained copolymers further exhibit temperature‐dependent solubility in aqueous solutions in the range from 30°C to 60°C, depending on the overall pyrenol content. Furthermore, the *T*_cloud_ can be increased by 3–5°C under blue light irradiation and by gradually increasing the solution pH until the copolymers are completely soluble. DLS and TEM experiments confirm the presence of nanoparticles in aqueous solution of P(EtOx_0.77_‐*co*‐PEI_0.06_‐*co*‐(HOPyr)SAz_0.17_), which could be tentatively explained by electrostatic attractions between HOPyr‐groups and PEI units due to partial proton transfer. Further DLS experiments show a decrease in the corresponding *R*_h_ in response to blue (405 nm) light exposure due to excited‐state proton transfer. It was shown for the first time that polymeric photoacids based on 1‐hydroxypyrene are non‐cytotoxic for several cell lines. Live cell imaging was able to confirm the ability of these copolymers to be taken up by the cells, further strengthening the observation that the observed non‐cytotoxic effects reflect a situation where the copolymers are internalized and also outside the cells. The fastest uptake rate (within 1 h) was observed for P(EtOx_0.77_‐*co*‐PEI_0.06_‐*co*‐(HOPyr)SAz_0.17_) and P(EtOx_0.78_‐*co*‐(HOPyr)SAz_0.22_) with the highest pyrenol content. Apart from that, P(EtOx_0.77_‐*co*‐PEI_0.06_‐*co*‐(HOPyr)SAz_0.17_) shows a high loading capacity of the model hydrophobic drug. In view of the above‐described outcome, poly(2‐oxazoline)‐based polyphotoacids have great potential as stimuli‐responsive drug carriers.

## 4 Experimental Section

### 4.1 Materials

1‐Hydroxypyren (>98%) was purchased from TCI and used as received. Triethylamine (TEA, ≥98%), chlorosulfonic acid (99%), methyl *p*‐toluenesulfonate (MeOTos, 98%) were purchased from Sigma Aldrich and used as received. Thionyl chloride (99.7%) was purchased from Acros Organics and used as received. Hydrochloric acid (∼37%, d = 1.18) was purchased from Fischer Scientific and used as received. 2‐Ethyl‐2‐oxazoline (EtOx, Sigma Aldrich, ≥99%) was dried over calcium hydride and distilled prior to use. Acetonitrile was purified by a solvent purification system SPS. All other chemicals were used as received.

### 4.2 Methods

#### 4.2.1 Nuclear Magnetic Resonance Spectroscopy

The ^1^H‐NMR measurements were conducted on the 300 MHz Bruker AC spectrometer (T = 298 K) using the residual solvent resonance as an internal standard. The chemical shifts are given in ppm. The compounds were directly dissolved in CDCl_3_, CD_3_OD, or DMSO‐d_6_ for the ^1^H‐NMR measurements.

#### 4.2.2 Size Exclusion Chromatography (SEC)

SEC was performed on an Agilent 1200 series system equipped with a G1310A pump, a G1315D DA detector, a G1362A RI detector, and PSS GRAM 30 Å/1000 Å (10 µm particle size, Polymer Standards Service GmbH, Mainz, Germany) columns in series at 40°C using *N,N*‐dimethylacetamide (DMAc) with 2.1 g/L LiCl as eluent at a flow rate of 1 mL/min. The system was calibrated using PEG standards (Mp = 400 to 1 000 000 g/mol). For SEC sample preparation, the polymers were directly dissolved in eluent (DMAc + 0.21 wt.% LiCl) and filtered by syringe filter (PTFE, 0.45 µm).

#### 4.2.3 UV/Vis Spectroscopy

UV/Vis measurements were conducted on an Agilent Cary 60 at room temperature, ranging from 250 to 800 nm. For the measurements, the polymers were directly dissolved in water at a concentration of 0.02 mg/mL. The samples' irradiation in solution was done by 200 W Hg(Xe) lamp from LOT‐QuantumDesign (Darmstadt, Germany) using a filter (U340 from Edmund Optics, Karlsruhe, Germany) coupled into a glass fiber, and placed over the sample.

The *pK*_a_* and Δ*pK*_a_ were obtained via Förster cycle analysis via the following equation [58]:

$$pK_{a}^{*}=pK_{a}\;-\frac{N_{a}hc}{2.303RT}*\left[\frac{1}{\lambda_{ROH}}-\frac{1}{\lambda_{RO^{-}}}\right]$$

where 𝑁_𝐴_ – Avagadro's number, ℎ – Plank's constant, 𝑐 – speed of light in vacuum, 𝑅 – universal gas constant, 𝑇 – solution temperature (298K), $\frac{1}{\lambda_{ROH}}$ and $\frac{1}{\lambda_{RO^{-}}}$ – 0,0 transition energy of ROH and RO^−*^ in cm^−1^ (determined by the mean of the energies of the lowest energy absorption and emission spectrum of ROH and RO^−*^, respectively).

The turbidity measurements were carried out in different heating ranges from 5°C to 70°C by comparing the absorbance at 800 nm. The 5 mg/mL polymer solutions were prepared by direct dissolving in water prior to the measurements. Irradiation of the samples was performed using a 405 nm LED source (M405L4, THORLABS), with irradiation time *t* = 2 h at a power *P* = 20 mW right before the measurements.

#### 4.2.4 Cytotoxicity Studies

A549 cells (5000 cells / well) or HEK293 cells (15 000 cells/well) were seeded into 96‐well plates and incubated overnight in cell culture media (Dulbecco's modified Eagle's medium (DMEM) (Sigma–Aldrich D6429); 10% fetal calf serum (Sigma–Aldrich F7524); 1% penicillin/streptomycin (P/S) (Sigma–Aldrich P0781)) at 37°C with 5% CO_2_. The cells were incubated with different concentrations of the polymers in DMEM with 1% P/S or only DMEM with 1% P/S as a negative control for 24 h. Subsequently, the biocompatibility of the polymers was investigated utilizing the CellTiter‐Blue Cell Viability Assay. The percentage of cell viability was calculated using the mean of the negative control, displaying 100% cell viability from at least three independent experiments using GraphPad Prism 10 software. According to DIN EN ISO 10993–5, a cell viability of ≤70% was considered toxic.

Furthermore, live‐cell imaging was applied to evaluate the cell morphology and the cellular polymer uptake. Therefore, A549 cells or HEK293 cells (at a density of 200 000 or 300 000 per well, respectively) were seeded onto sterile, poly‐D‐lysine‐coated 24 mm glass coverslips in cell culture media and incubated for 24 h at 37°C and 5% CO2. The cells were then incubated with 0.1 mg/mL of the polymers in DMEM with 1% P/S or only DMEM with 1% P/S as a control for 24 h. Images were acquired using an inverted laser scanning confocal microscope (DMi8 TCS SP8, Leica microsystems) equipped with a HC PL APO CS2 63x/1.20 WATER objective (Leica). Cells were sequentially excited first with a 442 nm diode laser and then a 405 nm UV laser, and emission in the range of 450–527 nm was sequentially collected using a hybrid detector. Bright‐field images were collected with a separate independent PMT detector. Images were later processed in ImageJ (https://imagej.nih.gov/ij/ NIH, Bethesda) to adjust and set identical contrast values over the entire collection of images for all polymers and time points.

#### 4.2.5 Live‐Cell Imaging to Monitor Polymer Uptake

HEK293 cells (at a density of 300 000 per well) were seeded onto 24 mm round poly‐D‐Lysine‐coated glass coverslips placed in a 6‐well culture plate and incubated in cell culture media at 37°C and 5% CO_2,_ and allowed to reach a confluency of over 70% over 48 h before imaging. For imaging, basal images of the cells were acquired before they were incubated separately with polymers at the indicated concentrations and times. Images were acquired as described above.

### 4.3 Synthesis

The synthesis of 1‐hydroxypyrene photoacid was performed according to the previously described method with minor modifications [52].

#### 4.3.1 6/8‐Hydroxypyrene‐1‐sulfonic Acid

1‐Pyrenol (3 g, 13.8 mmol) was dispersed in DCM (40 mL) and cooled to −10°C on an aceton/ice bath. Chlorosulphonic acid (1 mL, 15.1 mmol) was dissolved in DCM (20 mL) and added dropwise to the cold 1‐pyrenol solution under stirring. The obtained mixture was allowed to warm up to room temperature and stirred overnight. The DCM was removed in vacuum, and the residue was dispersed in diethyl ether and filtered. The filtrate was mixed with silica gel, evaporated, and purified on the flash chromatograph using DCM/methanol eluent, yielding the product as yellow–green solid. Yield = 1.2 g (30%). ^1^H NMR (300 MHz, DMSO‐d_6_):

6‐isomer: δ 9.04 (d, *J* = 9.6 Hz, Ar‐**H**, 1H), 8.46 (m, Ar‐**H**, 1H), 8.38 (m, Ar‐**H**, 1H), 8.18‐7.98 (m, Ar‐**H**, 4H), 7.88 (d, *J* = 8.9 Hz, Ar‐**H**, 1H), 7.62 (d, *J* = 8.3 Hz, Ar‐**H**, 1H) ppm.
8‐isomer: δ 8.91 (d, *J* = 9.3 Hz, Ar‐**H**, 1H), 8.47 (m, Ar‐**H**, 1H), 8.38 (m, Ar‐**H**, 1H), 8.18‐7.98 (m, Ar‐**H**, 4H), 7.63 (d, *J* = 8.3 Hz, Ar‐**H**, 1H) ppm.

#### 4.3.2 Sodium 6/8‐Acetoxypyrene‐1‐Sulphonate

The 1‐pyrenol sulphonate (1.2 g, 4 mmol) was dissolved in a methanol solution of NaOH (0.18 g, 4.5 mmol). The solvent was removed in vacuum, the residue was dispersed in acetic anhydride and stirred at 150°C for 2 h. The excess of acetic anhydride was removed under vacuum, and the residue was washed several times with diethyl ether and dried under reduced pressure, yielding the product as green‐gray powder. The sodium 6/8‐acetoxypyrene‐1‐sulfonate obtained by this procedure contains some amount of sodium acetate, which could be removed during the next steps. Yield = 1.3 g (94%). ^1^H NMR (300 MHz, DMSO‐d_6_):

6‐isomer: δ 9.23 (d, *J* = 9.6 Hz, Ar‐**H**, 1H), 8.54 (d, *J* = 8.0 Hz, Ar‐**H**, 1H), 8.39‐8.10 (m, Ar‐**H**, 5H), 7.90 (d, *J* = 8.2 Hz, Ar‐**H**, 1H), 2.57 (s, ─C(O)C**H_3_**, 3H) ppm.
8‐isomer: δ 9.17 (d, *J* = 9.2 Hz, Ar‐**H**, 1H), 8.55 (d, *J* = 7.9 Hz, Ar‐**H**, 1H), 8.39‐8.10 (m, Ar‐**H**, 5H), 7.90 (d, *J* = 8.2 Hz, Ar‐**H**, 1H), 2.56 (s, ─C(O)C**H_3_**, 3H) ppm.

#### 4.3.3 6/8‐Acetoxypyrene‐1‐Sulphonyl Chloride

6/8‐Acetoxypyrene‐1‐sulfonate synthesized at the previous stage, was added portion‐wise to the solution of thionyl chloride (10 mL, mol) in 50 mL of DCM under stirring. After adding, the reaction mixture was refluxed overnight. DCM and excess of thionylchloride were removed in vacuum, yielding a product as a green powder, which was used in the next steps without further purification.

^1^H NMR (300 MHz, DMSO‐d_6_):

6‐isomer: δ 9.23 (d, *J* = 9.6 Hz, Ar‐**H**, 1H), 8.54 (d, *J* = 8.0 Hz, Ar‐**H**, 1H), 8.39‐8.10 (m, Ar‐**H**, 5H), 7.90 (d, *J* = 8.3 Hz, Ar‐**H**, 1H), 2.57 (s, ─C(O)C**H_3_**, 3H) ppm.
8‐isomer: δ 9.17 (d, *J* = 9.4 Hz, Ar‐**H**, 1H), 8.55 (d, *J* = 8.0 Hz, Ar‐**H**, 1H), 8.39‐8.10 (m, Ar‐**H**, 5H), 7.90 (d, *J* = 8.3 Hz, Ar‐**H**, 1H), 2.56 (s, ─C(O)C**H_3_**, 3H) ppm.

#### 4.3.4 Homopolymerization of EtOx

The PEtOx homopolymer was synthesized via cationic ring‐opening polymerization (CROP) according to a standard procedure. In a glovebox, 2‐ethyl‐2‐oxazoline (EtOx) (8 mL, 79.4 mmol) and methyl 4‐methylbenzenesulfonate (MeOTos) (120 µL, 0.8 mmol) were dissolved in 11.8 mL of acetonitrile. The reaction mixture was stirred at 140°C for 16 min in the microwave synthesizer. The polymerization was terminated by adding 134 mg of NaN_3_ (3.2 mmol) and stirred overnight at 75°C. The obtained solution was precipitated into 400 mL of cold diethyl ether. The precipitate was dissolved in DCM, filtered, and the filtrate was evaporated in vacuum, yielding a product as a white powder.

#### 4.3.5 Partial Hydrolysis of PEtOx

PEtOx (0.5 g), was dissolved in 2.5 mL of water and mixed with 2.5 mL of a 37% solution of HCl. The reaction mixture was stirred on a magnetic stirrer at 75°C for indicated times (50, 100, 200, or 300 min). After the reaction, the pH value of the reaction mixture was adjusted to pH 11–12 by NaOH, and the water was removed by freeze‐drying. The residue was dissolved in DCM and filtered before the solvent was removed in vacuum, yielding the product as a white powder.

#### 4.3.6 General Procedure for the Functionalization of PEtOx‐co‐PEI with 6/8‐Acetoxypyrene‐1‐Sulphonyl Chloride (on the Example of P(EtOx_0.89_‐co‐(HOPyr)SAz_0.11_))

Partially hydrolyzed PEtOx‐*co*‐PEI (∼0.55 mmol of PEI units) obtained in the previous step was dissolved in 30 mL of DCM containing triethylamine (TEA, 0.31 mL, 2.2 mmol) and cooled on an acetone/ice bath to approx. −7°C. After that, the solution of 6/8‐acetoxypyrene‐1‐sulphonyl chloride (0.41 g, 1.1 mmol) in DCM (15 mL) was added dropwise. The reaction mixture was allowed to heat to room temperature and stirred overnight. Afterward, DCM was removed in vacuum, the residue was redissolved in methanol, filtered, and purified by preparative size exclusion chromatography (Sephadex LH20, eluent MeOH).

#### 4.3.7 General Procedure for the Deprotection of P(EtOx‐co‐(AcOPyr)SAz)

Guanidinium hydrochloride (0.5 g, 5.5 mmol) and 25 wt. % NaOMe solution in methanol (1.2 mL, 5.5 mmol) was dissolved in 10 mL of methanol and stirred for 10 min. The obtained solution was mixed with the polymer solution in methanol and stirred for another 6 h. Methanol was removed in a vacuum, the residue was redissolved in water, adjusted to pH 4–5 with HCl (37% in water), and dialyzed against water. The final copolymer was isolated by freeze‐drying as green‐yellow powder.

**Table:** 

Sample	Total yield, mg (%^a^)
P(EtOx_0.97_‐*co*‐(HOPyr)SAz_0.03_)	392 (78%)
P(EtOx_0.95_‐*co*‐(HOPyr)SAz_0.05_)	313 (63%)
P(EtOx_0.89_‐*co*‐(HOPyr)SAz_0.11_)	421 (75%)
P(EtOx_0.76_ *‐co*‐(HOPyr)SAz_0.24_)	280 (44%)
P(EtOx_0.77_‐*co‐PEI_0.06_‐co*‐(HOPyr)SAz_0.17_)	296 (46%)

^a^ Calculated based on *M*_n_ values determined by SEC (DMAc + 0.21 wt.% LiCl).

## Conflicts of Interest

The authors declare no conflicts of interest.

## Supporting information




**Supporting File**: marc70089‐sup‐0001‐SuppMat.docx.

## Data Availability

The data that support the findings of this study are available from the corresponding author upon reasonable request.
