# Supplementary material for: Poly(2‐Oxazoline)‐Based Polyphotoacids: Synthesis, Solution Behavior, and Cellular Uptake of Multi‐Responsive Intracellular Transporters
Source: Macromol Rapid Commun. 2025 Oct 16;47(14):e00455. doi: 10.1002/marc.202500455 (PMC13384808; doi:10.1002/marc.202500455)
Supplement: Supplementary file 1 — Supporting File: marc70089‐sup‐0001‐SuppMat.docx. [file MARC-47-e00455-s001.docx]

Supporting Information

**Poly(2-oxazoline)-based polyphotoacids: synthesis, solution behavior and cellular uptake of multi-responsive intracellular transporters**

*Leonid I. Kaberov,* Amod Godbole, Laura Klement, Sreevalsan Achikkulathu, Avinash Chettri, Benjamin Dietzek-Ivanšić, Carsten Hoffmann, and Felix H. Schacher*


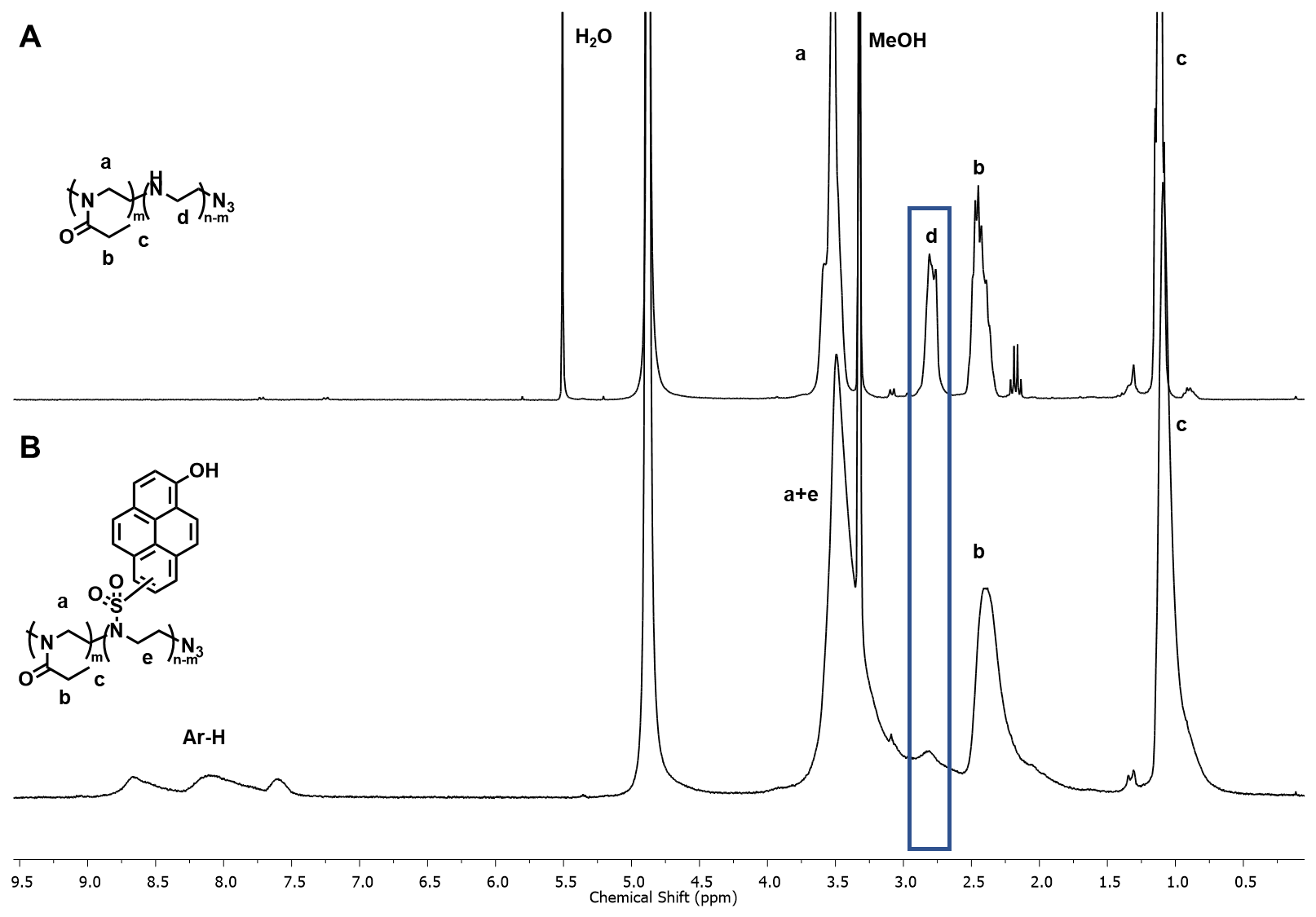


**Figure S1.** Stacked ^1^H-NMR of the P(EtOx_0.77_-*co*-EI_0.23_) (A), and P(EtOx_0.77_-*co*-PEI_0.06_-*co*-(HOPyr)SAz_0.17_) (B) copolymers in MeOD.

| P(EtOx_0.97_-*co*-(HOPyr)SAz_0.03_) | | |
| --- | --- | --- |
|  |  |  |
| P(EtOx_0.95_-*co*-(HOPyr)SAz_0.05_) | | |
|  |  |  |
| P(EtOx_0.89_-*co*-(HOPyr)SAz_0.11_) | | |
|  |  |  |
| P(EtOx_0.77_-*co-PEI*_0.06_*-co*-(HOPyr)SAz_0.17_) | | |
|  |  |  |

**Figure S2**. Steady-state absorption and emission spectra of obtained polyphotoacids in 4M HCl, pH 11.0 and pH 5.3. All experiments are conducted in non-inert conditions. The figures are adapted from previous work [57] please note that the value of the emission maximum of ROH* species provided in the main text has been wrongly reported earlier (Page 4, section “Steady-state absorption and emission spectroscopy ofHPSAPMA and I(a–d)”) - the correct value is $\lambda_{max}^{em.}$ = 445 nm, as determined from the emission spectra.

**
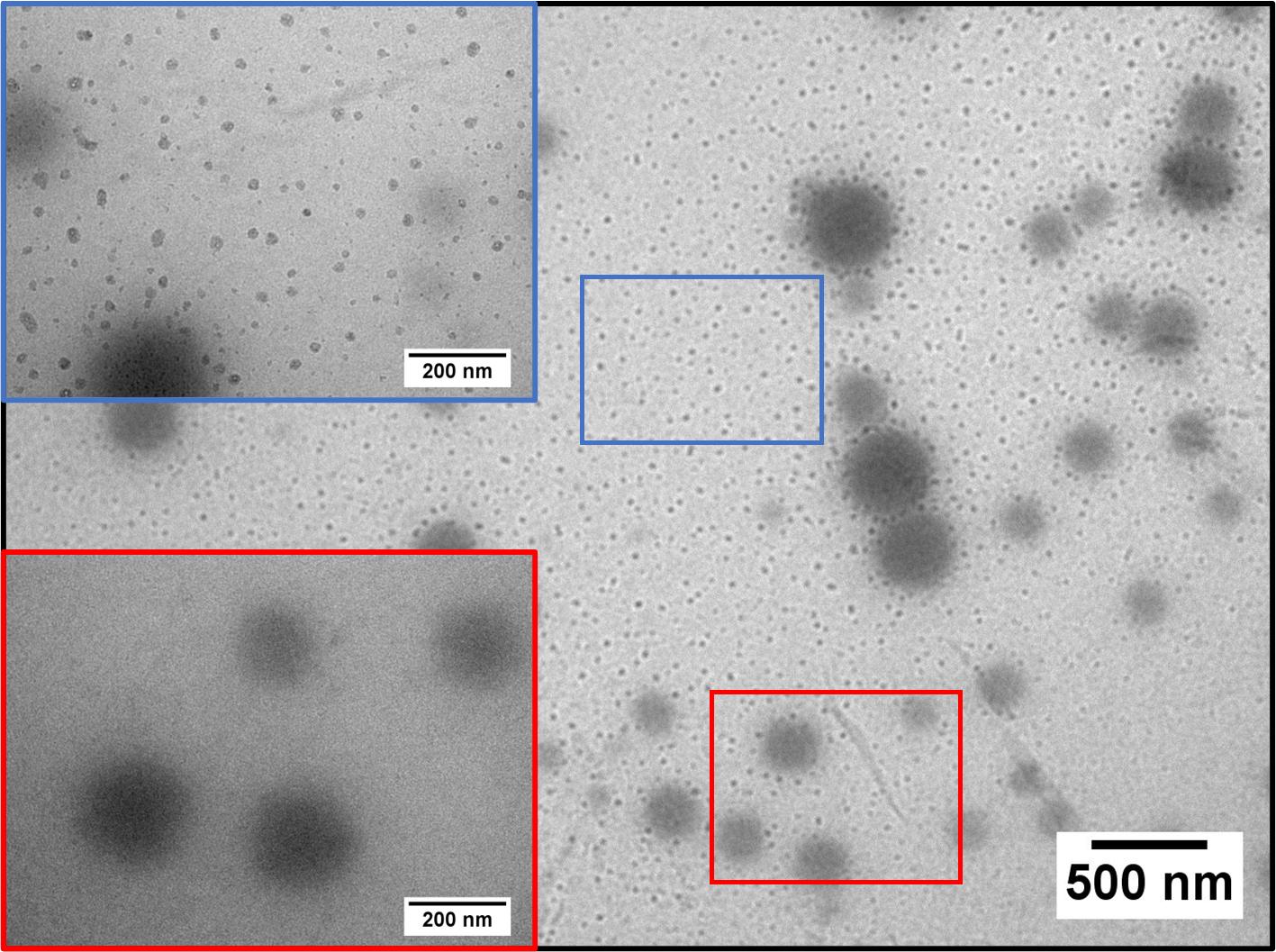
**

**Figure S3.** TEM micrograph of the nanoparticles formed by P(EtOx_0.77_-*co*-PEI_0.06_-*co*-(HOPyr)SAz_0.17_) in aqueous solution. C = 5 mg/mL.

**Figure S4.** UV vis spectra (left) and HPLC traces (right) of polyphotoacid, free curcumin, and curcumin loaded samples.

**Figure S5.** Transmittance vs. temperature plots for the P(EtOx_0.95_-*co*-(HOPyr)SAz_0.05_) polyphotoacids in aqueous solutions at different pHs. *C* = 1 mg/mL.


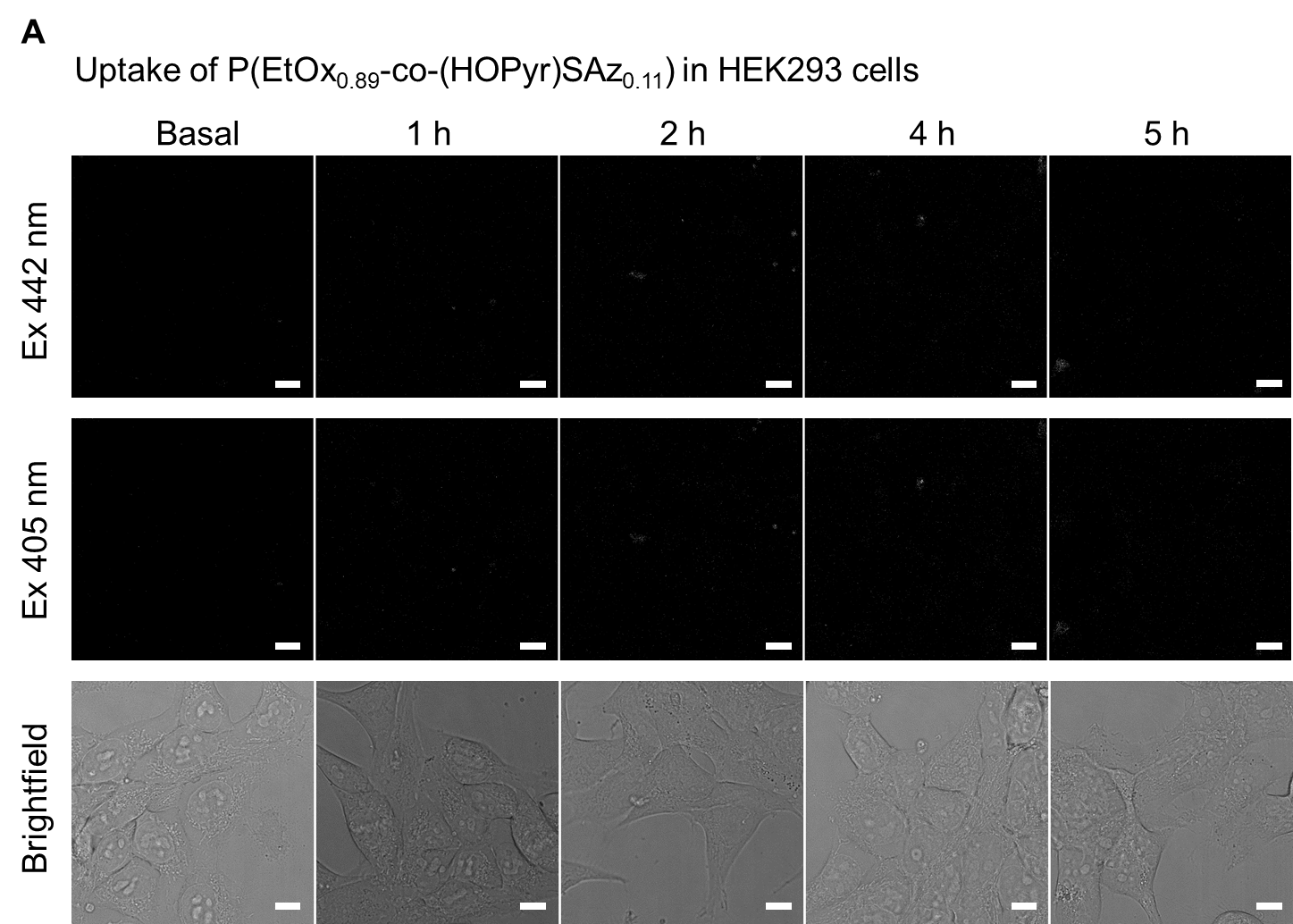


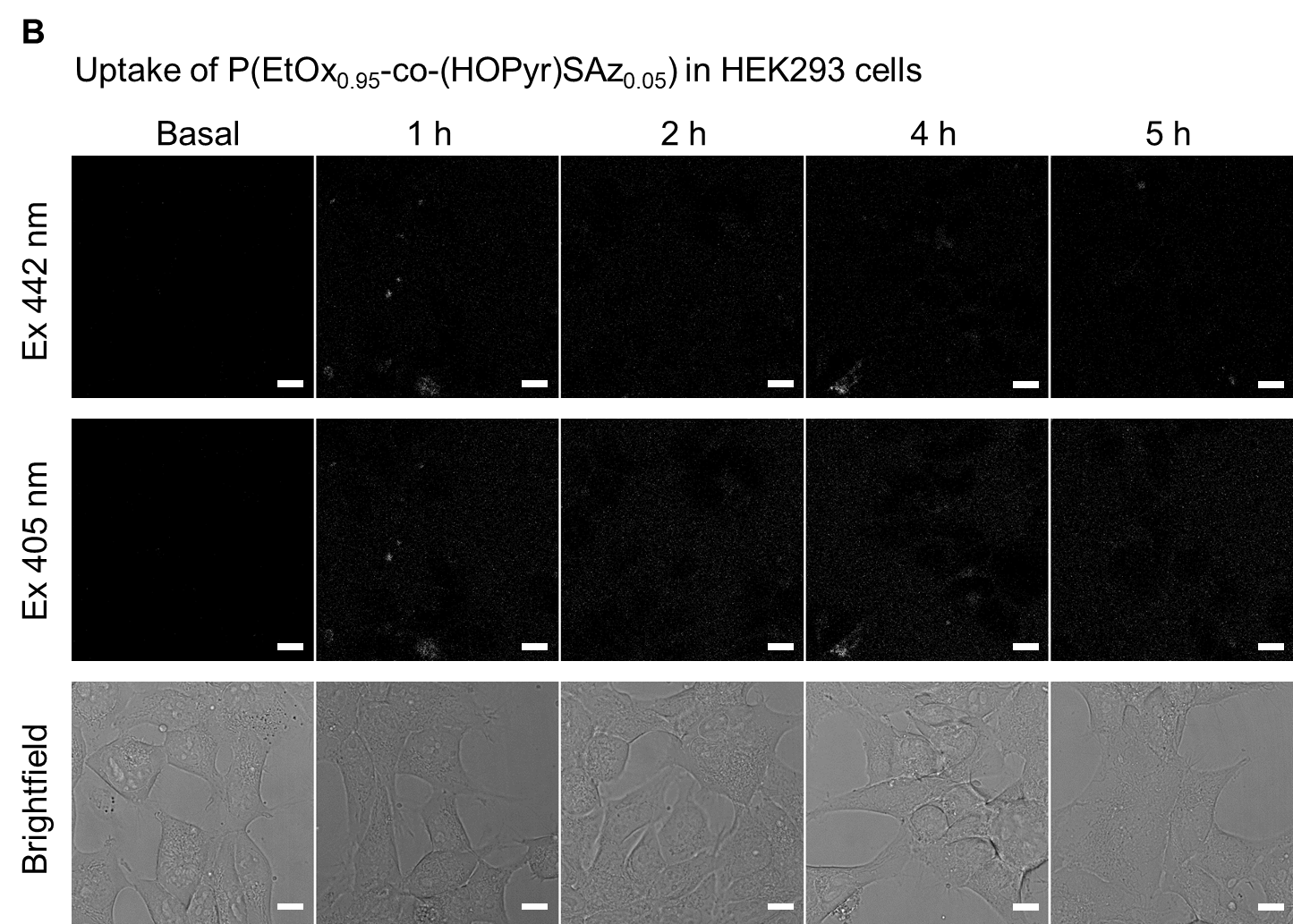


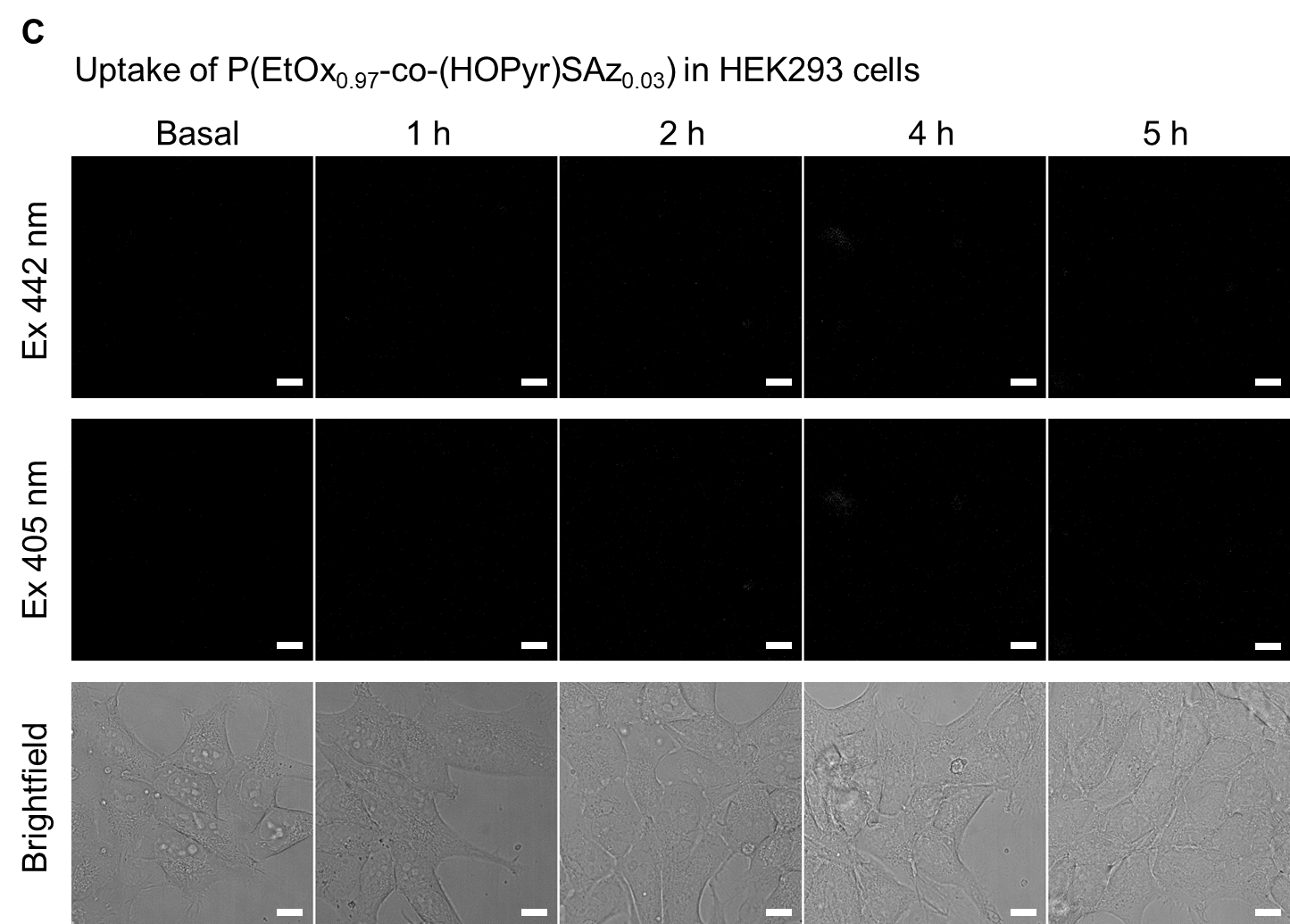


**Figure S6.** Uptake of polymers in living cells. Living HEK293 cells were incubated with P(EtOx_0.89_-*co*-(HOPyr)SAz_0.11_) (**A**), P(EtOx_0.95_-*co*-(HOPyr)SAz_0.05_) (**B**) or P(EtOx_0.97_-*co*-(HOPyr)SAz_0.03_) (**C**) polymers (with a final concentration of 0.1 mg/mL) in imaging buffer and images were acquired at indicated time points. Representative images with excitation at 442 nm (top panel), or 405 nm (middle panel) and emission in the range of 450-527 nm are shown here. Corresponding images using transmission light (bottom panel, Brightfield) were also acquired and shown here. The scale bar represents 10 µm.

**Table S1.** Cross-table of the samples labeling and compositions in the present and previous (Ref. 57, Chettri et. al, DOI: [**10.1002/chem.202401047**](https://doi.org/10.1002/chem.202401047)) work.

| **Composition, present work*** | **Composition, previous work**  **Chem. – A Eur. J.** | **Labelling**  **Chem. – A Eur. J.** |
| --- | --- | --- |
| P(EtOx_0.97_-*co*-(HOPyr)SAz_0.03_) | P(EtOx_0.97_-*co*-(HOPyr)SAz_0.03_) | **I (a)** |
| P(EtOx_0.95_-*co*-(HOPyr)SAz_0.05_) | P(EtOx_0.95_-*co*-(HOPyr)SAz_0.05_) | **I (b)** |
| P(EtOx_0.89_-*co*-(HOPyr)SAz_0.11_) | P(EtOx_0.89_-*co*-(HOPyr)SAz_0.11_) | **I (c)** |
| P(EtOx_0.76_*-co*-(HOPyr)SAz_0.24_) | Not presented | **-** |
| P(EtOx_0.77_-*co-PEI_0.06_-co*-(HOPyr)SAz_0.17_) | P(EtOx_0.78_-*co*-(HOPyr)SAz_0.22_) | **I (d)** |

*- Compositions, *M*_n_, and *Ð* values provided in the present work are preferred.
